# SOX17-mediated MALAT1-miR-199a-HIF1α axis confers sensitivity in esophageal squamous cell carcinoma cells to radiotherapy

**DOI:** 10.1038/s41420-022-01012-6

**Published:** 2022-05-25

**Authors:** Yifei Yun, Yutong Zhang, Qiqi Xu, Yao Ou, Xifa Zhou, Zhonghua Lu

**Affiliations:** Department of Radiotherapy, Changzhou Tumor Hospital, Soochow University, Changzhou, 213000 China

**Keywords:** Cancer, Cancer

## Abstract

Radiotherapy is a main modality of esophageal squamous cell carcinoma (ESCC) treatment, while radioresistance largely limits the effect of this therapy. Evidence exists reporting that SOX17 may sensitize ESCC cells to irradiation, but the downstream mechanism remains poorly understood. Therefore, we attempt to explore the molecular basis of SOX17 effect on radioresistance in ESCC. The SOX17 expression was measured in ESCC tissues and cells, followed by evaluation of its relationship with patient survival. The fractionated irradiation-induced irradiation-resistant cell line KYSE150R was subjected to gain- and loss-of function studies to explore the effect of SOX17 and downstream effectors MALAT1, miR-199a, and HIF1α on the malignant phenotypes of ESCC. The interaction among these factors was explained using ChIP, dual luciferase reporter, RNA pull-down and RIP assays. Further, the in vivo effect of SOX17 on ESCC irradiation tolerance was assessed in nude mice. SOX17 was underexpressed in ESCC tissues and cells, which was negatively correlated with the prognosis of patients with ESCC. Besides, SOX17 inhibited irradiation tolerance of ESCC cells by suppressing MALAT1 transcription. Notably, MALAT1 acted as miR-199a sponge and thereby enhanced HIF1α expression. Moreover, SOX17 reduced the irradiation tolerance of ESCC cells by reducing HIF1α expression via the MALAT1-miR-199a axis, and attenuated tumor formation in nude mice. Our results indicate that SOX17 can impede the radioresistance of ESCC cells through the MALAT1-miR-199a-HIF1α axis, in support of further research for ESCC radiotherapy.

## Introduction

Esophageal cancer is a common aggressive malignancy globally, which is listed as one of the four most fatal cancers in China, while esophageal squamous cell carcinoma (ESCC) is the major form of esophageal cancer around the world [1–3]. In the past few years, significant progress has achieved in the prevention and treatment of many cancers, but the overall 5-year survival rate of patients with ESCC is still below 20%, with the incidence of ESCC rapidly increasing worldwide [1]. Radiotherapy and surgical resection are the most important therapies for ESCC; however, the recurrence rates remain at high level by employing these managements [4]. The unsatisfactory outcomes may be caused by tumor radioresistance [5]. Therefore, an urgent need exists to explore the molecular mechanism of ESCC radioresistance and develop strategies to decrease the irradiation tolerance of ESCC cells.

SRY-box 17 (SOX17) transcription factor, an important member of high mobility group superfamily, is involved in various physiological processes [6]. Notably, emerging studies have provided evidence reporting the involvement of SOX17 in the pathogenesis of ESCC [7, 8]. For example, in cells and animal models, SOX17 poorly expresses in ESCC cells and tumor tissues; at the same time, overexpression of SOX17 could sensitize ESCC irradiation-resistant cells to irradiation, cisplatin and concurrent chemoradiation therapy treatment [9]. However, the effect of SOX17 on radioresistance and its related mechanisms still need further study.

Interesting, SOX17 could inhibit MALAT1 expression at the transcription level and subsequently affect the migration and invasion of ESCC cells by binding to the SRY element of the MALAT1 promoter region [10]. In addition, miRNAs (miRs) have become promising targets and tools for novel therapeutic approaches of cancers [11]. Thus, by bioinformatics analysis, we predict that several miRs, in the downstream of MALAT1, are related to ESCC in this study. Notably, among them, it has been demonstrated that miR-199a lowly expresses in esophagus cancer [12]. Following this, we further predicted the downstream regulatory factors of miR-199a, and found that hypoxia-inducible factor-1α (HIF1α), a putative target gene of miR-199a, significantly overexpresses in ESCC. Besides that, multiple studies have implied that inhibiting HIF1α could be a potential approach for ESCC treatment [13, 14], indicating that MALAT1 may upregulate the expression of HIF1α by targeting miR-199a and thereby mediate radioresistance.

Therefore, we hypothesized that SOX17 may affect the ESCC radioresistance by regulating MALAT1 expression and further modulating MALAT1-miR-199a-HIF1α axis, which may provide valuable strategies and targets for clinical management of ESCC.

## Results

### SOX17 is poorly expressed in ESCC and this low expression indicates poor prognosis of ESCC patients

**Fig. 1:**
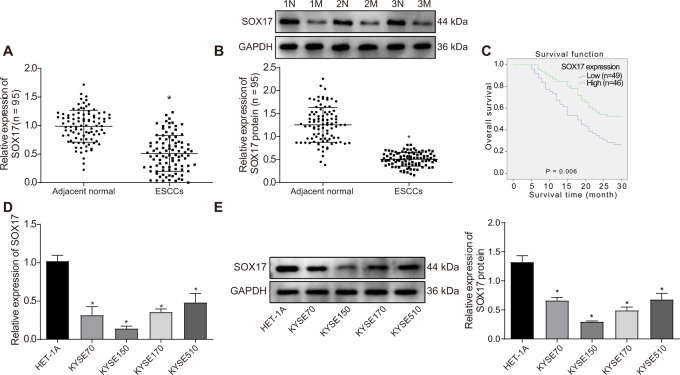
SOX17 expression was poorly expressed in ESCC tissues and cells, which was related to the prognosis of ESCC patients. **A**, **B** The expression of SOX17 mRNA and protein in ESCC tissues (ESCCs-Tumor group) and adjacent normal tissues (ESCCs-Adjacent normal group) of ESCC patients, measured by qRT-PCR and Western blot. *n* = 95 in ESCC patients; *p < 0.05 compared with adjacent normal tissues. N: ESCC adjacent normal tissues; M: ESCC tumor tissues. **C** Correlation between SOX17 expression and OS of ESCC patients analyzed by the Kaplan-Meier method. The expression of SOX17 mRNA and protein in human esophageal epithelial cell line HET-1A and ESCC cell lines KYSE70, KYSE170, KYSE150, and KYSE510 measured by qRT-PCR (**D**) and Western blot (**E**). *p < 0.05 compared with HET-1A cells. Measurement data were expressed as mean ± standard deviation. Paired *t* test was adopted to compare tumor tissues and adjacent normal tissues. The data of multiple groups were compared by one-way ANOVA, with Tukey’s post-hoc test. Cell experiments were repeated in triplicate.

First, we aimed to investigate the effect of SOX17 on the radioresistance of ESCC and its relatively downstream mechanism. As shown in Fig. 1A, B, the expression of SOX17 was lower in ESCC tissues than adjacent normal tissues. The overall survival (OS) of the patients with lower SOX17 expression was shorter (Fig. 1C), suggesting the association of low expression of SOX17 with poor prognosis of ESCC patients. In addition, the results of qRT-PCR and Western blot demonstrated that versus HET-1A cells, the expression of SOX17 was reduced in ESCC cell lines (KYSE70, KYSE170, KYSE150, and KYSE510), with KYSE150 cells presenting the lowest SOX17 expression (Fig. 1D, E) and thus selected for the experiments related to SOX17. The above results illustrated that SOX17 was underexpressed in ESCC tissues and cells, and this downregulation linked to the poor prognosis of ESCC patients.

### SOX17 inhibits irradiation tolerance of ESCC cells

**Fig. 2:**
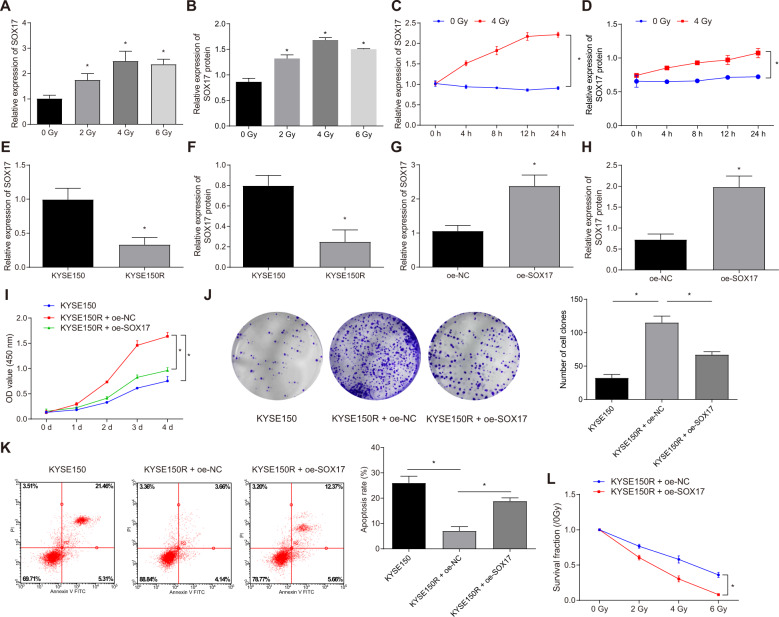
High expression of SOX17 could decrease the irradiation tolerance of ESCC cells. After treatment with different doses of X-ray, the mRNA and protein expression of SOX17 in KYSE150 cells was measured by qRT-PCR (**A**) and Western blot (**B**). *p < 0.05 compared with 0 Gy group. After exposure to a single dose of X-ray, the expression of SOX17 mRNA and protein in KYSE150 cells was examined by qRT-PCR (**C**) and Western blot (**D**). The expression of SOX17 mRNA and protein was measured by qRT-PCR (**E**) and Western blot (**F**) in KYSE150 cell line and its corresponding irradiation-resistant cell line KYSE150R. G-H, the mRNA and protein expression of SOX17 examined by qRT-PCR (**G**) and Western blot (**H**) in KYSE150R cells in response to oe-SOX17. I, Survival rates of KYSE150R cells in response to oe-SOX17 measured by MTT assay. **J** The proliferation ability of KYSE150R cells in response to oe-SOX17 examined by clonogenic assay **K** Flow cytometry was employed to evaluate the apoptosis of KYSE150R cells in response to oe-SOX17. **L** Clonogenic assay was adopted to measure the proliferation ability of KYSE150R cells in response to oe-SOX17. *p < 0.05. Measurement data were expressed as mean ± standard deviation. Data between two groups were analyzed by unpaired *t* test. Comparisons between multiple groups were performed by one-way ANOVA, followed by Tukey’s post-hoc test. Two-way ANOVA with Bonferroni post-hoc test was employed to compare the data of groups at different time points. Cell experiments were repeated in triplicate.

After measurement of qRT-PCR and Western blot, the results showed that the KYSE150 cells exposed to X-ray irradiation of higher dose presented with higher expression of SOX17 (Fig. 2A, B, Fig. S1A). Besides, no significant decline in the expression of SOX17 was measured when the X-ray irradiation dose reached 4 Gy and above, so the dose of 4 Gy was chosen for single dose irradiation. When a single dose of X-ray (4 Gy) was employed to irradiate KYSE150 cells, the expression of SOX17, examined by qRT-PCR and Western blot, was proportional to the exposure time (Fig. 2C, D, Fig. S1B). In comparison with that in KYSE150 cell line, the expression of SOX17 mRNA and protein in irradiation-resistant cell line KYSE150R was significantly lower (Fig. 2E, F, Fig. S1C). The above results demonstrated that SOX17 expression in ESCC cells was related to X-ray irradiation dose and time, and SOX17 expression was lower in irradiation-resistant cells, which may participate in the process of X-ray radiotherapy.

In order to investigate the impact of SOX17 on irradiation tolerance of KYSE150R cell line, we overexpressed SOX17 in KYSE150R cells. Relative to oe-NC group, the expression of SOX17 in KYSE150R cells was obviously higher in the oe-SOX17 group (Fig. 2G, H, Fig. S1D). MTT assay, clonogenic assay and flow cytometry results exhibited that after exposure to X-ray irradiation of 4 Gy, KYSE150R cells in oe-NC group showed higher survival rate (Fig. 2I), higher proliferation ability (Fig. 2J), and lower apoptosis (Fig. 2K), versus KYSE150 cells. In comparison with KYSE150R cells in oe-NC group, KYSE150R cells in oe-SOX17 group displayed lower survival rate (Fig. 2G), lower proliferation ability (Fig. 2J), and higher apoptosis (Fig. 2K) after treatment with X-ray irradiation of 4 Gy. After exposure KYSE150R cells in oe-SOX17 group and oe-NC group to different doses of X-ray, the result of clonogenic assay showed that KYSE150R cells in the oe-SOX17 group exposed to irradiation of higher dose displayed with faster decrease of cell proliferation. (Fig. 2L, Fig. S2A), suggesting that KYSE150R cells had increased sensitivity and reduced tolerance to X-ray irradiation through overexpressing SOX17 in KYSE150R cells.

The above results indicated that high expression of SOX17 could reduce the irradiation tolerance of ESCC cells.

### SOX17 reduces irradiation tolerance of ESCC cells by transcriptional inhibition of MALAT1

SOX17 could inhibit the transcription of MALAT1 by binding to SRY component in MALAT1 promoter region in the context of ESCC, and further affect the invasion and migration of ESCC cells [10]. Therefore, in the next studies, we investigated whether SOX17 could affect the irradiation effect of ESCC cells by suppressing the expression of MALAT1.

**Fig. 3:**
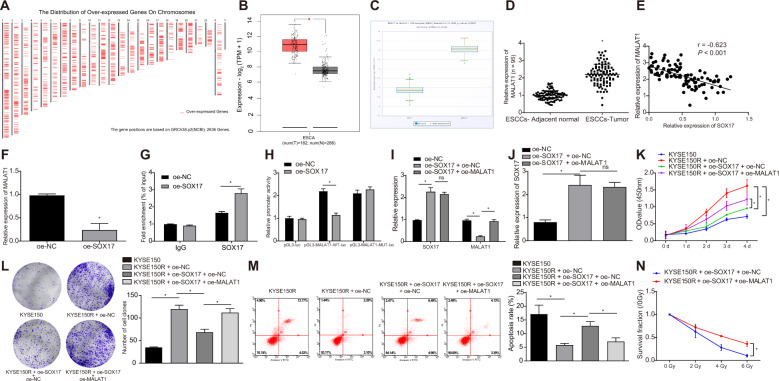
SOX17 suppressed the radioresistance of ESCC cells via transcriptional inhibition of MALAT1. **A** Chromosome map showing location of 2636 DEGs which were significantly overexpressed in ESCC. **B** A box plot of MALAT1 expression in normal samples and ESCC samples. Red box represents cancer samples, and gray box represents normal samples. **C** The co-expression relationship between SOX17 and MALAT1 in ESCC samples. D, MALAT1 expression in tumor tissues (ESCC-Tumor group) and adjacent normal tissues (ESCC-Adjacent normal group) from ESCC patients as measured by qRT-PCR. *n* = 95 in ESCC patients. **E** Correlation analysis of MALAT1 expression and SOX17 mRNA expression in tumor tissues of 95 ESCC patients analyzed by Pearson correlation analysis. **F** The expression of MALAT1 in KYSE150R cells in oe-NC group and oe-SOX17 group as examined by qRT-PCR. **G** MALAT1 binding to SOX17 in KYSE150R cells of oe-NC group and oe-SOX17 group measured by ChIP assay. **H** The luciferase activity of MALAT1 promoter in 293T cells of oe-NC group and oe-SOX17 group measured by dual luciferase reporter assay. **I** The expression of SOX17 and MALAT1 in KYSE150R cells in response to oe-SOX17 alone or combined with oe-MALAT1 examined by qRT-PCR. **J** The protein expression of SOX17 in KYSE150R cells in response to oe-SOX17 alone or combined with oe-MALAT1 measured by Western blot. K, The survival rate of KYSE150R cells in response to oe-SOX17 alone or combined with oe-MALAT1 measured by MTT assay. **L** The proliferation ability of KYSE150R cells in response to oe-SOX17 alone or combined with oe-MALAT1 examined by clonogenic assay. **M** The apoptosis of KYSE150R cells in response to oe-SOX17 alone or combined with oe-MALAT1 measured by flow cytometry. **N** The proliferation ability of KYSE150R cells in response to oe-SOX17 alone or combined with oe-MALAT1 examined by clonogenic assay. *p < 0.05. Measurement data were expressed as mean ± standard deviation. Paired *t* test was employed to compare the difference between tumor tissues and adjacent normal tissues. Unpaired *t* test was adopted to analyze the other data between two groups. One-way ANOVA followed by Tukey’s post-hoc test was used for comparison between multiple groups. Two-way ANOVA with Bonferroni post-hoc test was employed to analyze the data between groups at different time points. Cell experiments were repeated in triplicate.

Based on bioinformatics tool GEPIA, we obtained 2636 significantly overexpressed genes by genetic difference analysis of ESCC samples in the TCGA database (Fig. 3A). After sorting the expression of differentially expressed genes (DEGs) related to ESCC, we found the differential expression of MALAT1 was the most evident presenting with the lowest *p* value (Table S1), and MALAT1 was highly expressed in ESCC (Fig. 3B). Thus, MALAT1 was selected for further studies. Chipbase v2.0 website was adopted to analyze the co-expression between SOX17 and MALAT1 in ESCC (Fig. 3C). qRT-PCR results showed higher expression of MALAT1 in tumor tissues than that in adjacent normal tissues (Fig. 3D). In addition, MALAT1 expression was adversely correlated to SOX17 expression in tumor tissues (Fig. 3E).

After overexpressing SOX17 in KYSE150R cells, MALAT1 was significantly underexpressed, as measured by qRT-PCR (Fig. 3F). In addition, we revealed that MALAT1 promoter which was binding to SOX17 evidently increased through ChIP assay (Fig. 3G) Moreover, the luciferase activity of MALAT1 promoter significantly decreased following SOX17 overexpression, as detected by luciferase reporter (Fig. 3H). The above results suggested that SOX17 could bind to the promoter region of MALAT1 to restrict the transcription of MALAT1 in ESCC cells.

The expression of SOX17 was higher in oe-SOX17 + oe-NC group than oe-NC group (Fig. 3I, J, Fig. S1E). Besides, in comparison with that in oe-NC group, MALAT1 expression was inhibited in oe-SOX17 + oe-NC group. Additionally, MALAT1 expression was elevated in the oe-SOX17 + oe-MALAT1 group versus the oe-SOX17 + oe-NC group (Fig. 3I). Versus oe-SOX17 + oe-NC group, the KYSE150R cells in oe-SOX17 + oe-MALAT1 group had higher survival rate (Fig. 3K), promoted cell proliferation (Fig. 3L), and lowered apoptosis (Fig. 3M).

When KYSE150R cells of the oe-SOX17 + oe-NC group and the oe-SOX17 + oe-MALAT1 group were exposed to X-ray irradiation at different doses, the decrease in the proliferation of KYSE150R cells in oe-SOX17 + oe-MALAT1 group was impeded with higher irradiation dose, as examined by clonogenic assay (Fig. 3N, Fig. S2B). This result illustrated that simultaneous overexpression of SOX17 and MALAT1 reversed the promoting effect of SOX17 overexpression on the radiosensitivity of ESCC cells.

Overall, the above results indicated that SOX17 suppressed the irradiation tolerance of ESCC cells through transcriptional inhibition of MALAT1

### MALAT1 competitively binds to miR-199a

**Fig. 4:**
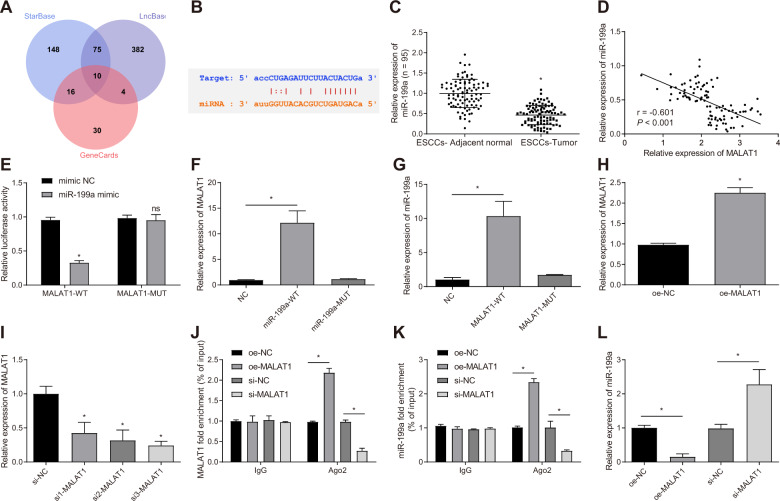
MALAT1 competitively bound to miR-199a in ESCC cells. **A** Venn diagram of downstream miRs of MALAT1 predicted by starBase and LncBase databases and the ESCC-related miRs obtained from the GeneCards database. **B** The binding site of MALAT1 and miR-199a predicted by starBase website. **C** The expression of miR-199a in tumor tissues (ESCCs-Tumor group) and adjacent normal tissues (ESCCs-Adjacent normal group) in ESCC patients, as detected by qRT-PCR. *n* = 95 in ESCC patients. **D** Correlation analysis of miR-199a expression and MALAT1 expression in tumor tissues of 95 ESCC patients analyzed by Pearson correlation analysis. **E** Dual luciferase report assay was adopted to examine the binding affinity between MALAT1 and miR-199a. **F** RNA-pull down assay was employed to detect the binding between biotin-labeled miR-199a-WT with MALAT1, and the binding between miR-199a-MUT with MALAT1. **G** RNA-pull down assay was employed to detect the affinity between the biotin-labeled MALAT1-WT and miR-199a, and the affinity between the MALAT1-MUT with miR-199a. **H** The expression of MALAT1 in KYSE150R cells was measured by qRT-PCR in oe-NC group and oe-MALAT1 group. **I** qRT-PCR was employed to detect the transfection efficiency of si-MALAT1 in KYSE150R cells. **J** RIP experiment was employed to assess the enrichment of MALAT1 in Ago2 in KYSE150R cells in response to oe-MALAT1 or si-MALAT1. **K** RIP experiment was employed to assess the enrichment of miR-199a in Ago2 in KYSE150R cells in response to oe-MALAT1 or si-MALAT1. **L** The expression of miR-199a was examined in KYSE150R cells in response to oe-MALAT1 or si-MALAT1 examined by qRT-PCR. *p < 0.05 compared with adjacent normal tissues, mimic-NC group, NC group, oe-NC group or si-NC group. ns, not significant. Measurement data were presented as mean ± standard deviation. Paired *t* test was employed to analyze the data of tumor tissues group and adjacent normal tissues group. The other two groups were compared by unpaired *t* test. One-way ANOVA with Tukey’s post-hoc test was employed to compare the data of multiple groups. Cell experiments were repeated in triplicate.

In order to further explore the downstream regulatory mechanism of MALAT1 affecting ESCC cell radioresistance, we first predicted the downstream miRNAs of MALAT1 using starBase and LncBase databases. Besides, through the GeneCards database, we found 60 miRs related to ESCC, which were then intersected with the above predicted miRs, with 10 candidate miRs obtained (Fig. 4A). Among them, miR-199a was previously reported to be poorly expressed in ESCC [12], and thus we selected miR-199a for further study.

starBase website predicted the binding site of MALAT1 and miR-199a (Fig. 4B). qRT-PCR data revealed poor miR-199a expression in ESCC tissues versus normal adjacent tissues (Fig. 4C). A negative correlation was found between miR-199a expression and MALAT1 expression in ESCC tissues (Fig. 4D). The luciferase activity of MALAT1-WT in the miR-199a mimic group was decreased while no alteration was found in the MALAT1-MUT luciferase activity (Fig. 4E). In the RNA-pull down experiment, the biotin-labeled miR-199a-WT probe could significantly pull down MALAT1, and the biotin-labeled MALAT1-WT probe could markedly pull down miR-199a (Fig. 4F, G). These data confirmed that MALAT1 could bind to miR-199a.

MALAT1 was overexpressed in KYSE150R cells (Fig. 4H). Three si-MALAT1 sequences were applied to knock down MALAT1 gene in KYSE150R cells, and the expression of MALAT1 in KYSE150R cells was measured by qRT-PCR. As shown in Fig. 4I, the expression of MALAT1 was reduced after transfection of si-MALAT1, with si3-MALAT1 showing the superior efficiency and thus selected for the following experiments.

By RIP detection, MALAT1 and miR-199a were significantly enriched in Ago2 in KYSE150R cells with oe-MALAT1 while sh-MALAT1 reduced the enrichment (Fig. 4J, K). In KYSE150R cells, the expression of miR-199a was reduced in the presence of MALAT1 overexpression while it was increased after MALAT1 silencing (Fig. 4L).

Taken together, MALAT1 could competitively bind to miR-199a in ESCC cells.

### MALAT1 upregulates HIF1α expression by competitively binding to miR-199a

**Fig. 5:**
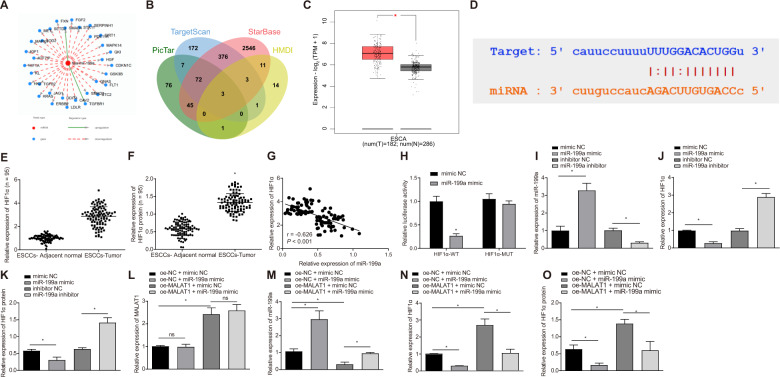
MALAT1 enhanced HIF1α expression through binding to miR-199a. **A** Network diagram of target genes of miR-199a based on bioinformatics website HMDD. **B** Venn diagram of the target genes of miR-199a predicted by HMDD, PicTar, TargetScan and starBase websites. **C** A box plot of HIF1α expression in ESCC samples. **D** The binding site of miR-199a and HIF1α predicted by the starBase website. **E**, **F** The expression of HIF1α mRNA and protein in tumor tissues (ESCC-Tumor group) and adjacent normal tissues (ESCC-Adjacent normal group), as measured by qRT-PCR and Western blot. *n* = 95 in ESCC patients. **G** Correlation analysis of miR-199a expression and HIF1α mRNA expression in tumor tissues of 95 ESCC patients analyzed by Pearson correlation analysis. **H** Dual luciferase reporter assay was employed to examine the binding between miR-199a and HIF1α mRNA. **I** The miR-199a expression was examined by qRT-PCR in KYSE150R cells in response to miR-199a mimic or miR-199a inhibitor. The mRNA and protein expression of HIF1α measured by qRT-PCR (**J**) and Western blot (**K**) in KYSE150R cells in response to miR-199a mimic or miR-199a inhibitor. **L** The MALAT1 expression measured by qRT-PCR in KYSE150R cells in response to miR-199a mimic and oe-MALAT1 alone or in combination. **M** The miR-199a expression in KYSE150R cells in response to miR-199a mimic and oe-MALAT1 alone or in combination examined by qRT-PCR assay. The mRNA and protein expression of HIF1α in KYSE150R cells in response to miR-199a mimic and oe-MALAT1 alone or in combination examined by qRT-PCR (**N**) and Western blot (**O**). *p < 0.05, compared with adjacent normal tissues, mimic NC group, inhibitor NC group, oe-NC + mimic NC group, or oe-MALAT1 + mimic NC group. Measurement data were showed as mean ± standard deviation. Data between tumor tissues and adjacent normal tissues were compared by paired *t* test. The other two group comparison was analyzed by unpaired *t* test. Data of multiple groups were compared by one-way ANOVA with Tukey’s post-hoc test. Cell experiments were repeated three times.

HMDD website was employed to analyze the network diagram of target genes mediated by miR-199a (Fig. 5A). From the intersection of miR-199a target genes predicted by HMDD, PicTar, TargetScan and starBase databases, we obtained three candidate genes SIRT1, GSK3B and HIF1A (Fig. 5B). Among them, the expression of HIF1α in ESCC increased more significantly (Fig. 5C). The binding site of miR-199a and HIF1α was obtained using starBase website (Fig. 5D). The expression of HIF1α was higher in ESCC tissues than that in adjacent normal tissues (Fig. 5E, F, Fig. S1F). Pearson correlation analysis revealed an adverse correlation between miR-199a and HIF1α in ESCC tissues (Fig. 5G).

The luciferase activity of HIF1α-WT in the miR-199a mimic group was repressed while that of HIF1α-MUT was almost unchanged (Fig. 5H). KYSE150R cells were treated with miR-199a mimic and miR-199a inhibitor (Fig. 5I). The mRNA and protein expression of HIF1α decreased evidently after miR-199a overexpression in KYSE150R cells, and it increased markedly after miR-199a inhibition (Fig. 5J, K, Fig. S1G). The above results illustrated that miR-199a could target HIF1α and further reduce its expression.

Compared with oe-NC + mimic NC group, miR-199a expression was significantly higher in oe-NC + miR-199a mimic group. Meanwhile, in oe-MALAT1 + mimic NC group, the MALAT1 expression increased and the miR-199a expression diminished (Fig. 5L, M). Relative to oe-NC + mimic NC group, expression of HIF1α was inhibited in oe-NC + miR-199a mimic group, while it was highly expressed in oe-MALAT1 + mimic NC group. In addition, compared with oe-MALAT1 + mimic NC group, the expression of HIF1α mRNA and protein was significantly lower in oe-MALAT1 + miR-199a mimic group (Fig. 5N, O, Fig. S1H).

Collectively, MALAT1 could upregulate the expression of HIF1α by binding to miR-199a in ESCC cells.

### SOX17 suppresses HIF1α through the MALAT1-miR-199a axis and thus reduces the irradiation tolerance of ESCC cells

**Fig. 6:**
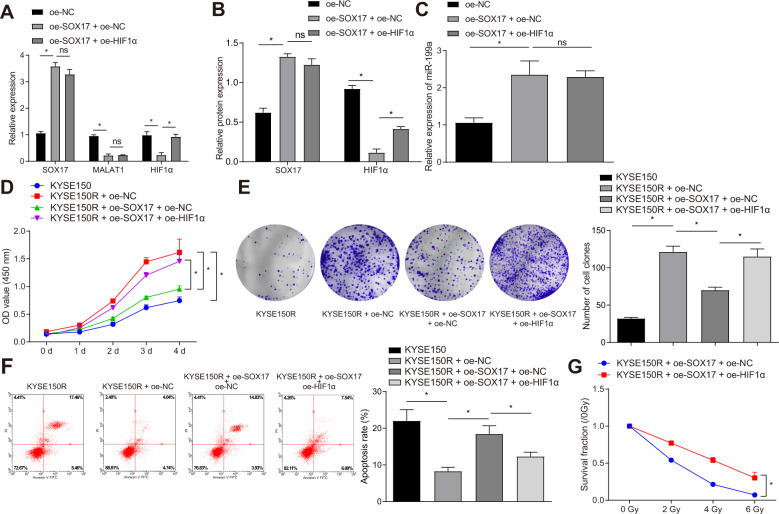
SOX17 inhibited HIF1α expression by modulating the MALAT1-miR-199a axis and thus reduced the resistance of ESCC cells to irradiation. **A** The expression of SOX17, HIF1α and MALAT1 in KYSE150R cells in response to oe-SOX17 alone or combined with oe-HIF1α measured by qRT-PCR. **B** The protein expressions of SOX17 and HIF1α in KYSE150R cells in response to oe-SOX17 alone or combined with oe-HIF1α examined by Western blot. **C** The expression of miR-199a in KYSE150R cells in response to oe-SOX17 alone or combined with oe-HIF1α measured by qRT-PCR. **D** The survival rate of KYSE150R cells in response to oe-SOX17 alone or combined with oe-HIF1α examined by MTT assay. **E** The proliferation ability of KYSE150R cells in response to oe-SOX17 alone or combined with oe-HIF1α assessed by clonogenic assay. **F** The apoptosis of KYSE150R cells in response to oe-SOX17 alone or combined with oe-HIF1α evaluated by flow cytometry. **G** The proliferation ability of KYSE150R cells in response to oe-SOX17 alone or combined with oe-HIF1α measured by clonogenic assay. *p < 0.05, ns, not significant. Measurement data were presented as mean ± standard deviation. One-way ANOVA with Tukey’s post-hoc test was employed to analyze data of multiple groups. Two-way ANOVA followed by Bonferroni post-hoc test was adopted to compare the data among multiple groups at different time points. Cell experiments were repeated for three times.

Next, we aimed to examine whether SOX17 affects the radioresistance of ESCC cells by regulating the MALAT1/miR-199a/HIF1α axis. qRT-PCR and Western blot results showed that the mRNA and protein expression of SOX17 in the oe-SOX17 + oe-NC group was promoted, while the expression of MALAT1 was obviously lowered (Fig. 6A, B, Fig. S1I). Meanwhile, miR-199a expression was overexpressed in the oe-SOX17 + oe-NC group (Fig. 6C), while the mRNA and protein expression of HIF1α was decreased. Versus oe-SOX17 + oe-NC group, we found no difference in the expression of SOX17, MALAT1 and miR-199a in the oe-SOX17 + oe-HIF1α group, while HIF1α expression markedly restored.

Compared with oe-SOX17 + oe-NC group, the survival rate and proliferation of KYSE150R cells were enhanced after 4 Gy X-ray exposure in the oe-SOX17 + oe-HIF1α group (Fig. 6D, E), and the apoptosis was obviously lower (Fig. 6F). In comparison with oe-SOX17 + oe-NC group, a halted decrease was observed in the proliferation ability of KYSE150R cells with higher X-ray dose in the oe-SOX17 + oe-HIF1α group (Fig. 6G, Fig. S2C). This result illustrated that after simultaneously overexpressing SOX17 and HIF1α in KYSE150R cells, the sensitivity of KYSE150R cells to X-ray irradiation decreased accompanied by an enhanced tolerance, thus HIF1α reversed the effects of SOX17.

These results revealed that SOX17 overexpression could inhibit HIF1α through the MALAT1-miR-199a axis and thus reduced the irradiation tolerance of ESCC cells.

### SOX17 overexpression inhibits radioresistance of ESCC by downregulating HIF1α in vivo

**Fig. 7:**
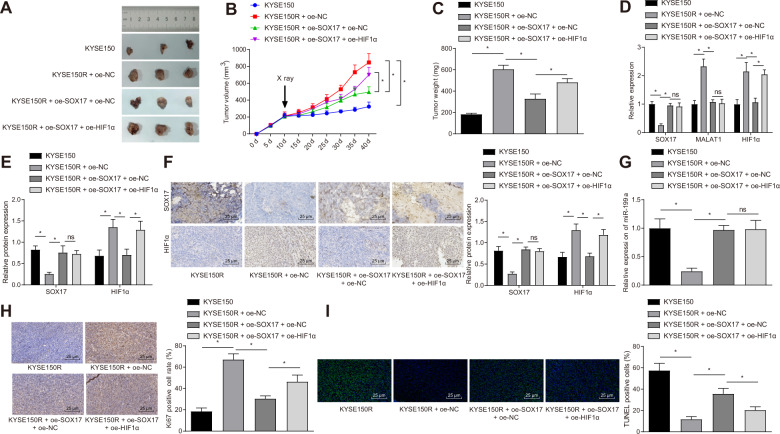
Overexpression of SOX17 inhibited the irradiation tolerance of ESCC by mediating the MALAT1/miR-199a/HIF-1α axis in vivo. Nude mice were divided into KYSE150, KYSE150 + oe-NC, KYSE150 + oe-SOX17 + oe-NC and KYSE150 + oe-SOX17 + oe-HIF1α groups. **A** Representative images of tumors of nude. **B** Quantitative analysis for tumor volume of nude mice. **C** Quantitative analysis for tumor weight of nude mice. **D** SOX17 and HIF1α mRNA expression in tumor tissues of mice as measured by qRT-PCR. **E** The protein expression of SOX17 and HIF1α in tumor tissues of mice, as examined by Western blot analysis. **F** The protein expression of SOX17 and HIF1α in tumor tissues of mice measured by immunohistochemistry **G** The miR-199a expression in tumor tissues of mice measured by qRT-PCR. **H** The expression of ki67 in tumor tissues of mice examined by immunohistochemistry. **I** Apoptosis of tumor cells in tumor tissues of mice measured by TUNEL assay. *p < 0.05 compared with the KYSE150 group, or the KYSE150 + oe-NC group. Measurement data were presented as mean ± standard deviation. Data of multiple groups were compared by one-way ANOVA with Tukey’s post-hoc test. Data of multiple groups at different time points were analyzed by two-way ANOVA, followed by Bonferroni post-hoc test. *n* = 12 for nude mice in each group.

KYSE150 cells and KYSE150R cells were inoculated into nude mice, and these mice were exposed to 4 Gy X-ray, followed by weight and tumor size measurement. After exposing to X-ray irradiation, compared with KYSE150 group, the tumorigenicity of nude mice was enhanced in the KYSE150R + oe-NC group (Fig. 7A–C). Versus KYSE150R + oe-NC group, the tumorigenicity of nude mice reduced in KYSE150R + oe-SOX17 + oe-NC group after X-ray irradiation (Fig. 7A–C). Meanwhile, relative to KYSE150R + oe-SOX17 + oe-NC group, the tumorigenicity of mice was promoted in KYSE150R + oe-SOX17 + oe-HIF1α group after X-ray irradiation (Fig. 7A–C). As shown in Fig. 7D–G and Fig. S1J, the expression of SOX17 and miR-199a was downregulated and that of HIF1α and MALAT1 was upregulated in tumor tissues of mice in the KYSE150R + oe-NC group. However, KYSE150R + oe-SOX17 + oe-NC group showed reversed results in comparison to KYSE150R + oe-NC group.

In addition, HIF1α expression was higher in the KYSE150R + oe-SOX17 + oe-HIF1α group than that in KYSE150R + oe-SOX17 + oe-NC group. At the same time, the results of immunohistochemistry and TUNEL staining showed increased cell proliferation and decreased cell apoptosis in the KYSE150R + oe-NC group, but opposite results were found in the KYSE150R + oe-SOX17 + oe-NC group. In addition, the KYSE150R + oe-SOX17 + oe-HIF1α group exhibited higher cell proliferation and lower cell apoptosis than the KYSE150R + oe-SOX17 + oe-NC group (Fig. 7H, I).

These results showed that SOX17 overexpression could reduce the HIF1α expression via transcriptional repression of MALAT1 and upregulation of miR-199a, thus delaying the irradiation tolerance of ESCC cells in vivo.

## Discussion

Radiotherapy is an effective approach for ESCC treatment [15] but radiation tolerance often occurs, limiting the clinical application of radiotherapy in a large extent [16]. Interestingly, accumulating evidence shows that SOX17, expressed at low levels in many different types of cancers [17, 18], is associated with the radiotherapeutic sensitivity of ESCC [9]. Moreover, SOX17 could affect the invasion and migration of ESCC cells by inhibiting the expression of MALAT1 [10]. In addition, related evidence also shows that miRs are crucial regulators to radiosensitivity [19], which may have roles to play in the radioresistance of ESCC cells by mediating downstream target genes. However, few studies have focused on the influence of SOX17 on ESCC radioresistance, and the mechanisms still remain poorly understood. Therefore, in this investigation, we illustrated that SOX17 had an impact on the ESCC radioresistance by suppressing MALAT1 expression and further regulating the expression of its downstream miR-199a and HIF1α.

Our experimental results showed that SOX17 was underexpressed in ESCC cells and tissues, and SOX17 overexpression could reduce the irradiation tolerance of ESCC cells. SOX17 has been reported as an important transcription factor involved in different physiological processes [6], and may be a valuable biomarker for various cancers such as ESCC [7], colorectal cancer [20], and breast cancer [17]. In addition, Kuo et al. also demonstrated that SOX17 overexpression could sensitize chemoradiation response in ESCC [9]. Moreover, SOX17 could bind to MALAT1 promoter region and thus suppress MALAT1 expression [10], which implies that the effect of SOX17 on the irradiation sensitivity of ESCC may be related to MALAT1. So, after overexpressing SOX17 or MALAT1 in irradiation-resistant ESCC cells, we demonstrated that SOX17 could reduce the irradiation tolerance through transcriptional inhibition of MALAT1.

Accumulating evidence suggests that miRs and their biogenesis mechanism are involved in the process of cancer development [11], and multiple miRs have become potential treatment strategies or targets of therapeutics [21–23]. For example, miR-455-3p and miR-296 may play crucial roles in ESCC progression and provide potential therapeutic targets for better clinical outcomes [24, 25]. Through bioinformatics analysis, we screened several downstream regulators of MALAT1, which were also associated with ESCC. Among them, miR-199a is expressed at low levels in esophageal cancer [12]. Further, our experimental results showed that MALAT1 could act as a miR-199a sponge, indicating that the SOX17 effect on radioresistance of ESCC cells may be related to the MALAT1-miR-199a axis.

Subsequently, we sought to uncover the target genes regulated by miR-199a. By predicting the regulatory factors of miR-199a based on the bioinformatics websites, we obtained three candidate genes, including SIRT1, GSK3B and HIF1α, of which HIF1α is more highly and significantly expressed in ESCC cells. Notably, several researches have revealed that HIF1α inhibition could suppress tumor growth of ESCC and sensitize ESCC cells to therapeutic approaches [13, 14, 26]. For example, Dihydroartemisinin might sensitize ESCC cells to photodynamic therapy by inhibiting the HIF1α pathway [14]. Moreover, mitochondrial pyruvate carrier blocker UK5099 could activate HIF1α expression, and UK5099-treated ESCC cells showed obviously more resistant to irradiation, as well as higher invasive ability in comparison with the parental cells [27]. Similarly, in the current research, we also demonstrated that SOX17 could suppress the HIF1α expression through the MALAT1-miR-199a axis to reduce the radioresistance of ESCC cells. In addition, we further illustrated that in the model of nude mice, SOX17 overexpression could inhibit irradiation tolerance of ESCC by downregulating HIF1α.

In conclusion, our experimental results showed that SOX17 inhibited MALAT1 expression at the transcription level, and thus potentiated the irradiation sensitivity of ESCC cells. Mechanistically, MALAT1 upregulated HIF1α by sponging miR-199a. SOX17 could prevent the irradiation tolerance of ESCC through the MALAT1-miR-199a-HIF1α axis, which may provide potential targets against ESCC radioresistance.

## Materials and methods

### Ethics statement

The current study was performed in strict accordance with the *Declaration of Helsinki*. All experimental protocols have been approved by the Ethics Committee of Changzhou Tumor Hospital, Soochow University. All patients have signed informed consent. The procedures of animal assay were in compliance with *Guide for the Care and Use of Laboratory Animals* published by the National Institutes of Health.

### Study subjects

From December 2014 to January 2017, 95 patients (54 males and 41 females, aged 40–70 years, a mean age of 58.57 ± 6.92 years), who were pathologically confirmed as ESCC after operation, were enrolled in this study. The part in the central of cancer tissue without bleeding around necrosis and the normal mucosa, 2 cm away from the distal esophagus, were collected as resected specimens. Patients received no radiotherapy or chemotherapy before surgery. The clinicopathological features of 95 patients with ESCC are shown in Table S2. All the subjects were followed up and their conditions and clinical outcomes post treatment were recorded in detail. The follow-up period was 3–30 months, from the end of surgery to July 2019. The relationship between gene expression and OS of patients was determined using the Kaplan-Meier method.

### Bioinformatics analysis

DEGs in ESCC samples in TCGA database were analyzed using GEPIA database, with |logFC| > 1 and *p* value < 0.01 as the threshold. Differentially expressed lncRNAs were selected, and the co-expression relationships between SOX17 and DEGs in esophageal cancer were analyzed by Chipbase v2.0. Downstream miRs of lncRNAs were predicted using starBase and LncBase databases. Meanwhile, miRs related to esophageal cancer were retrieved from GeneCards database and then intersected with the downstream miRs of lncRNAs using jvenn tool. Through the HMDD, PicTar, TargetScan and starBase websites, the downstream target genes of miRs were predicted, and then intersected using jvenn tool. Candidate genes were screened based on their expression in esophageal cancer.

### RNA extraction and qRT-PCR

Total RNA from tissues and cells was extracted using TRIzol (Invitrogen, Carlsbad, CA). TaqMan MicroRNA Assay RT primer (4427975, Applied Biosystems) or PrimeScript RT Reagent Kit (RR047A, Takara, Japan) were employed to synthesize the cDNA from mRNA. qRT-PCR assay was conducted by Thermo’s TaqMan Multiplex Real-Time Solution (4461882) and ABI 7500 real-time PCR system, with primer sequences shown in Table S3. The expression of targets was calculated by 2^−△△CT^, normalized to U6 or GAPDH.

### Western blot

Tissues and cells were lysed with RIPA buffer and the BCA kit was employed to determine protein concentration in the protein extract. The protein was separated and transferred onto membranes. The membranes were incubated with primary antibodies (rabbit anti-SOX17, 1:500, Abcam, Cambridge, UK; rabbit anti-HIF1α, 1:5000, Sigma). The membranes were then incubated with horseradish peroxidase-labeled anti-rabbit IgG (1:1000, Santa Cruz Biotech, CA). Immunoblots were visualized and captured using Bio-Rad ChemiDoc™ imaging system. GAPDH (rat, 1:1000, Santa Cruz Biotech, CA) was used as an internal reference, and the protein band image was analyzed by ImageJ2x software.

### Cell culture and transfection

Human ESCC cell lines KYSE70, KYSE170, KYSE150, and KYSE510 were all purchased from American Type Culture Collection (Manassas, VA, USA), and the normal esophageal epithelial cell line HET-1A was obtained from Oulu Biotechnology (Guangzhou, Guangdong, China). They were all maintained in RPMI-1640 medium (Gibco, Waltham, MA) with 10% fetal bovine serum (FBS; Gibco), 100 μg/mL streptomycin and 100 μg/mL penicillin. Cells were cultured in a 5% CO_2_ incubator at 37 °C.

Human ESCC radiotherapy-resistant cell line KYSE150R was established by fractionated X-ray irradiation [28]. KYSE150 cells were cultured in RPMI-1640 medium (Gibco, Carlsbad, CA) containing 10% FBS (Hyclone, Logan) in a humidified incubator with 5% CO_2_ (v/v). After KYSE150 cells had grown to 50–60% density, they were treated with 2 Gy X-ray irradiation (2.5 Gy/min) using a Varian-6/100 linear accelerator (Varian Medical Systems, Inc, Palo Alto, CA). Subsequently, the KYSE150 cell culture medium was changed to RPMI-1640 medium containing 10% FBS, and the medium was changed every 2 days. When many dead cells were observed, 15% FBS-containing RPMI-1640 medium was used for cell culture. When the irradiated cells reached 70–80% confluence, the above irradiation process was repeated until the total radiation dose reached 60 Gy. Finally, the obtained cells were named KYSE150R cells and continued to be cultured for ≥2 weeks before subsequent experiments.

Cells were trypsinized and plated into six-well plates (1 × 10^5^ cells/well). After 24 h of routine culture, when cells reached 50% confluence, transient transfection was performed using Lipofectamine 2000 (Invitrogen). The experimental groups included oe-NC, oe-SOX17, oe-SOX17 + oe-NC, oe-SOX17 + oe-MALAT1, oe-MALAT1, si-NC (5′-AUUGUAUGCGAUCGCAGACUU-3′), siRNA targeting MALAT1 (si-MALAT1) [si1-MALAT1 (forward: 5′-GCAAAUGAAAGCUACCAAU-3′, reverse: 5′-AUUGGUAGCUUUCAUUUGCTT-3′), si2-MALAT1 (forward: 5′-GCAGAGGCAUUUCAUCCUU-3′, reverse: 5′-AAGGAUGAAAUGCCUCUGCTT-3′), si3-MALAT1 (forward: 5′-CACAGGGAAAGCGAGTGGTTGGTAA-3′, reverse: 5′-TTACCAACCACTCGCTTTCCCTGTG-3′)], mimic NC, miR-199a mimic, inhibitor NC, miR-199a inhibitor, oe-NC + mimic NC, oe-MALAT1 + mimic NC, oe-NC + miR-199a mimic, oe-MALAT1 + miR-199a mimic and oe-SOX17 + oe-HIF1α groups. Transfection plasmids, mimic and inhibitor were all purchased and synthesized in Sino Biological Inc. (Beijing, China). After the transfected cells were cultured for 6 h, the medium was renewed. After incubation for 48 h, the cells were collected and used for subsequent experiments.

### Cell viability assay

After ESCC cells in each group were exposed to X-ray irradiation, the cell culture plate was centrifuged for 5 min, and then the old medium in the well was replaced with MTT (1 mg/mL) dissolved the fresh medium without light exposure. After that, the culture plate was incubated for 3 h, and centrifuged for 6 min. MTT solution was discarded from the well and 100 µL DMSO was added. The absorbance was detected by a microplate reader at 450 nm.

### Clonogenic assay for cell proliferation

ESCC cells were digested with 0.25% trypsin and pipetted to single cells (1 × 10^6^ cells/mL). Cells (500 cells/well) were seeded into 24-well plates containing 1 mL of 37 °C preheated culture medium and incubated at 37 °C with 5% CO_2_ for 2–3 weeks, with medium renewed every 2–3 days. Next, the cells fixed by 5 mL of 4% paraformaldehyde and incubated for 15 min. The cells were stained with GIEMSA (Invitrogen) for 10–30 min and air-dried. The colonies were photographed and counted with an inverted microscope (DMi8-M, Leica Co. Ltd., Solms, Germany), and colonies with more than 50 cells were considered as effective clones. Planting efficiency (%) = (effective clone number/plated cell number) × 100%; Survival fraction (%) = (Plant efficiency of radiation treatment group/Plant efficiency of control group) × 100%.

### Flow cytometry

ESCC cells were seeded into six-well plates (2 × 10^5^ cells/well) Experimental groups were classified into blank group, negative control group and transfection group. After transfection at 100 nmol/L and incubation for 72 h, the cells were trypsinized and centrifuged in a 15 mL centrifuge tube. Afterwards, as per the instructions of AnnexinV-FITC Apoptosis Detection Kit Ι (BD Biosciences, San Jose, CA), the cells were resuspended in 500 μL binding buffer, and incubated with 5 μL FITC and 5 μL PI in the dark for 15 min. Cell apoptosis was measured by a FACSCalibur flow cytometer (BD Biosciences).

### RNA-pull down assay

Biotinylated probe MALAT1-WT and mutant control MALAT1-Mut were designed and synthesized by RiBo Biological Co., Ltd (RiBo, Guangzhou, China). ESCC cells were lysed in lysis buffer and respectively incubated with 3 μg MALAT1-WT or MALAT1-Mut for 2 h. The mixture was incubated with magnetic beads for 4 h to pull down the biotin-coupled RNA complex. TRIzol reagent was employed to extract the miRs bound in the pull-down complex. The miR-199a levels in the pull-down samples were assessed by qRT-PCR. Biotin-labeled probe miR-199a-WT and mutant miR-199a-Mut were both designed and synthesized in RiBo Biological Co., Ltd (RiBo, Guangzhou, Guangdong China). The pull-down assay was carried out as described above. The expression of MALAT1 was detected by qRT-PCR.

### ChIP assay

ESCC cells were fixed in 1% formaldehyde solution and incubated at 37 °C for 10 min, followed by addition of glycine solution. The cells were lysed in 200 μL SDS lysis buffer, sonicated to generate chromatin fragments and immunoprecipitated with antibodies to SOX17 (Rabbit, 2 μg for 25 μg of chromatin, Abcam, Shanghai, China), and IgG (Rabbit, Abcam, serving as NC). Precipitated protein-DNA complex was eluted and cross-linking was reversed. DNA fragments were purified and analyzed by qRT-PCR.

### Luciferase activity assay

Reporter plasmids containing MALAT1-WT and MALAT1 mutated at the putative miR-199a binding site were inserted into Pmir-GLO Dual-Luciferase miRNA Target Expression Vectors (Promega, Madison, WI). Similarly, reporter plasmids containing HIF1α-WT and HIF1α-MUT at the miR-199a binding site were inserted into the luciferase reporter vector. These reporter plasmids were co-transfected with mimic NC and miR-199a mimic into 293T cells. After 48 h of transfection, luciferase activity was measured by the luciferase assay kit (K801-200, BioVision, Mountain View, CA) and the dual luciferase reporter assay system (Promega, Madison, WI), with Renilla luciferase an internal reference.

Reporter plasmids containing pGL3-luc, pGL3-MALAT1-WT-luc and pGL3-MALAT1-MUT-luc in the 3′UTR of SOX17 were inserted into the pGL3 Luciferase Reporter Vectors (Promega) and then co-transfected with oe-NC and oe-SOX17 into 293T cells (Oulu Biotechnology). Activity of MALAT1 promoter was detected.

### RIP assay

RIP assay was performed using Magna RIP Kit (Millipore, Billerica, MA). Cells were collected and lysed with 100 μL RIP lysis buffer with protease inhibitor and ribonuclease inhibitor. Next, the cell lysate was incubated with anti-Ago2 (1 μg, Rabbit, Abcam) and protein A/G-beads (30 μL). The samples were digested with proteinase K to isolate the immunoprecipitated RNA. The purified RNA was subjected to qRT-PCR.

### Subcutaneous tumor growth experiments

Forty-eight 5-week-old SPF male BALB/c nude mice (18–22 g, Shanghai SLAC Laboratory Animal Co., Ltd. (Shanghai China) were used for tumorigenesis experiments. The mice were anesthetized with ether and routinely disinfected. KYSE150 cells (1 × 10^6^ cells/200 μL) in oe-NC group, oe-SOX17 + oe-NC group and oe-SOX17 + oe-HIF1α group were subcutaneously injected into the dorsal surface of the mouse right hind limb. Tumor volume was measured every 5 days. When the average tumor volume grew to 200 mm^3^, the mice were irradiated with 4 Gy X-ray for 5 consecutive days. When the average tumor diameter of the mice in the oe-NC group without irradiation treatment reached 1.5 cm, the mice were euthanized by cervical dislocation.

### Immunohistochemistry

The tumor tissue sections (5 μm) were subjected to antigen retrieval, blocked with 1% BSA for 1 h, and incubated with primary antibody (rabbit anti-HIF1α, 1:500, rabbit anti-ki67, 1:100; Sigma) overnight at 4 °C and then with HRP-conjugated IgG (Boster, Wuhan, China) for 1 h. Further, the sections were exposed to DAB (Boster) for development. After counterstained with hematoxylin, the sections were dehydrated and subjected to microscopic examination after mounting.

### TUNEL assay

TUNEL assay was conducted as per the manufacturer’s instructions (MILLIPORE-SIGMA, St. Louis, MO). TUNEL-positive cells with brown nuclear staining were counted under an Olympus microscope by researchers who were blinded to the treatment allocation of this study.

### Statistical analysis

Statistical comparison was performed using paired *t* test or unpaired *t* test when only two groups were compared, or by Tukey’s test-corrected one-way ANOVA when more than two groups were compared. Two-way ANOVA with Bonferroni’s post-hoc test was employed to compare multi-group data at different time points. Data were presented as mean ± SD. All statistical analyses were completed with SPSS 21.0 software with *p* < 0.05 as a level of statistical significance.

## Supplementary information

Supplemental Materials

Supplemental Materials - original western blots
