# Supplementary material for: SOX17-mediated MALAT1-miR-199a-HIF1α axis confers sensitivity in esophageal squamous cell carcinoma cells to radiotherapy
Source: Cell Death Discov. 2022 May 25;8:270. doi: 10.1038/s41420-022-01012-6 (PMC9132944; doi:10.1038/s41420-022-01012-6)
Supplement: Supplementary file 1 — Supplemental Materials [file 41420_2022_1012_MOESM1_ESM.doc]

**Supplemental Materials**

**
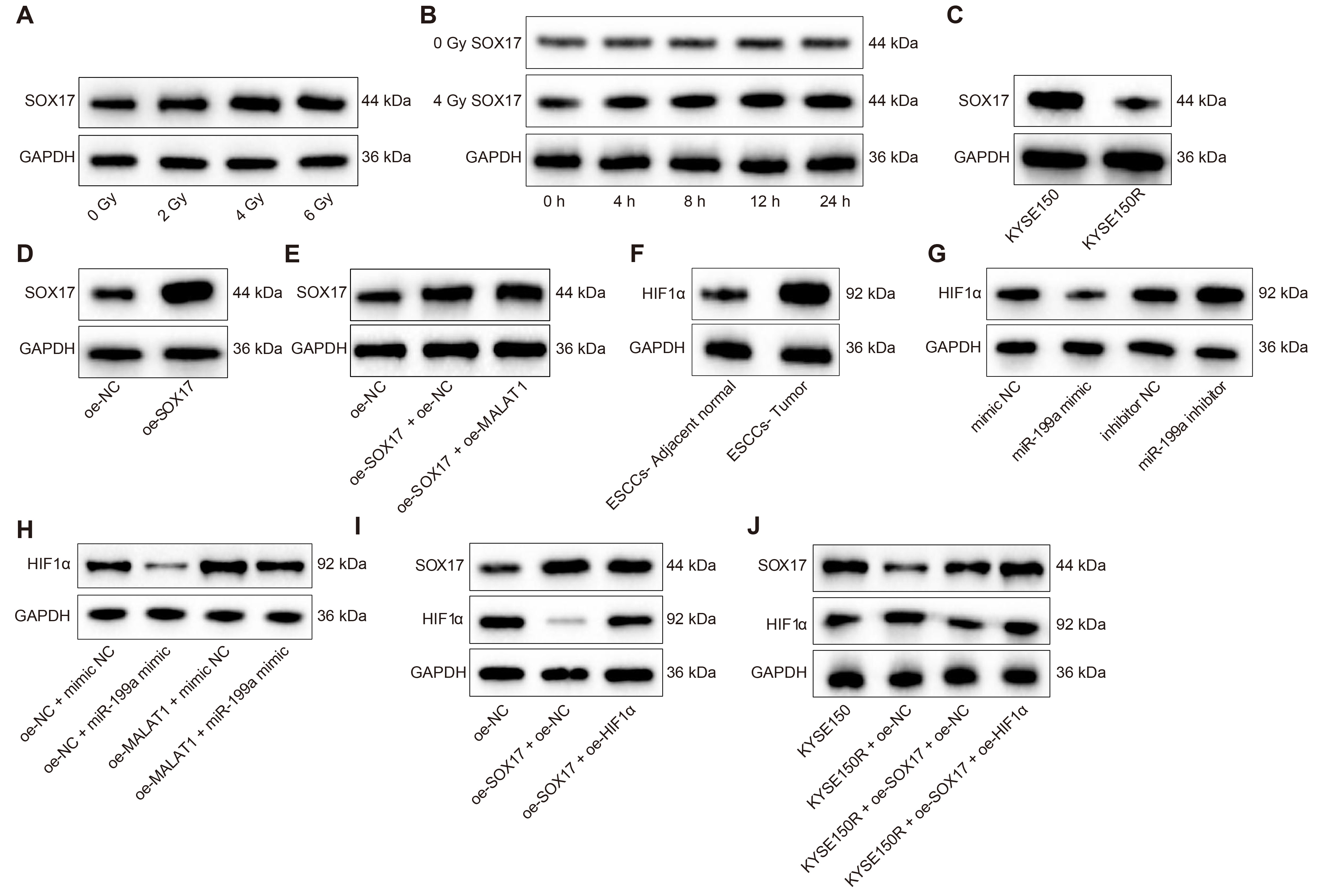
**

**Figure S1 |** Representative Western blots for quantification of protein expression in Figure 2B (A), 2D (B), 2F (C), 2H (D), 3J (E), 5F (F), 5K (G), 5O (H), 6B (I), and 7E (J).

**
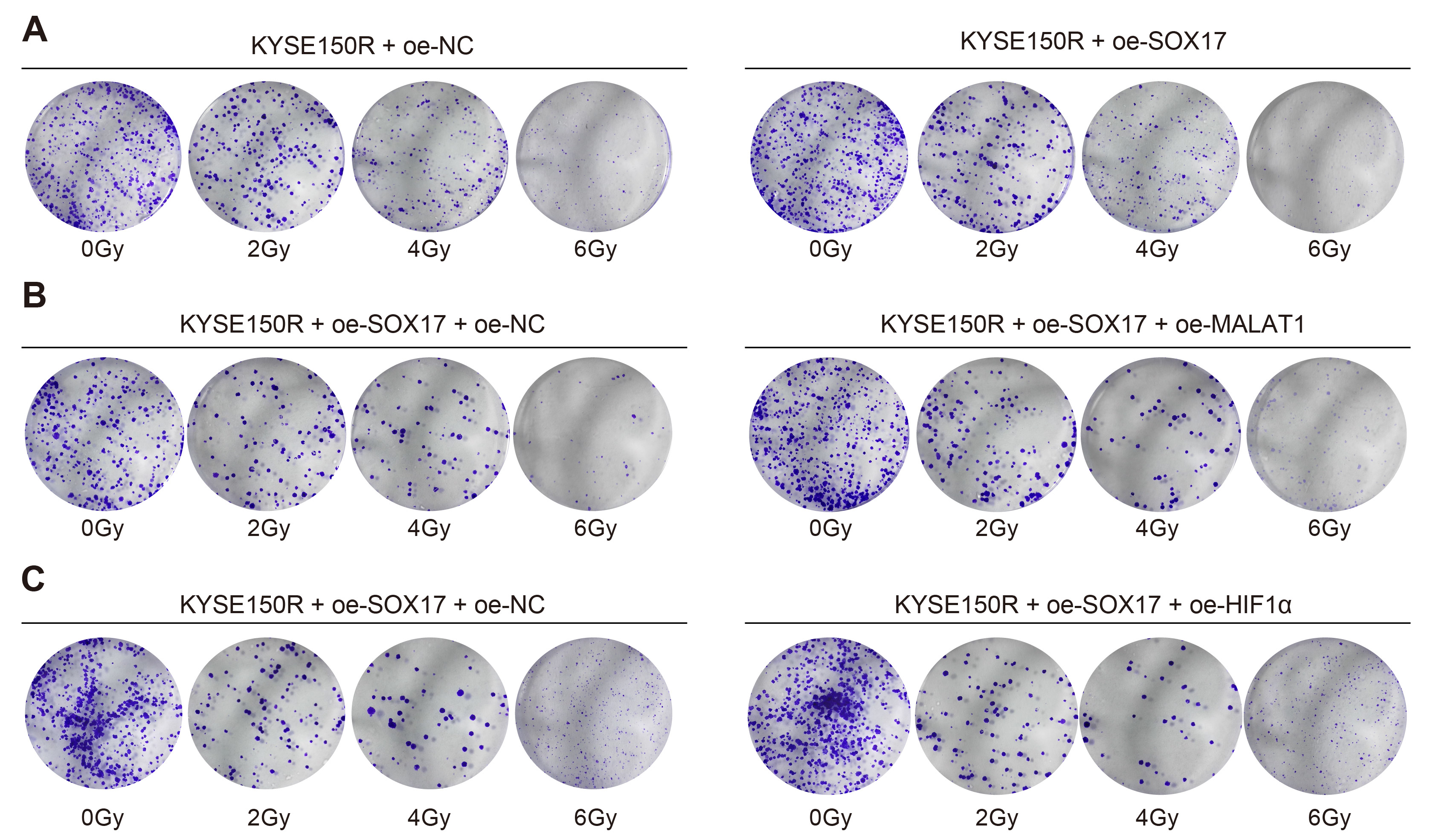
**

**Figure S2 |** Representative images of clonogenic assay for quantification of proliferation ability of KYSE150R cells in Figure 2L (A), 3N (B), and 6G (C).

**Table S1 |** Differential gene expression related to esophageal squamous cell carcinoma (ESCC)

| Gene Symbol | Median (Tumor) | Median (Normal) | Log2 (Fold Change) | adjp |
| --- | --- | --- | --- | --- |
| B2M | 5315.169 | 1660.036 | 1.678 | 3.27E-26 |
| CH507-513H4.3 | 3850.152 | 430.568 | 3.158 | 1.94E-48 |
| CH507-513H4.4 | 3850.152 | 430.568 | 3.158 | 1.94E-48 |
| CH507-513H4.6 | 3850.152 | 430.568 | 3.158 | 1.94E-48 |
| MT-ND3 | 3606.165 | 7538.699 | -1.064 | 6.29E-19 |
| FTL | 2693.935 | 964.273 | 1.481 | 2.19E-31 |
| PABPC1 | 2623.995 | 1107.813 | 1.243 | 5.61E-32 |
| IGKC | 2573.203 | 535.131 | 2.263 | 2.18E-13 |
| MALAT1 | 1970.983 | 176.576 | 3.473 | 1.51E-70 |

Note: B2M, beta-2-microglobulin; CH507-513H4.3, uncharacterized CH507-513H4.3; CH507-513H4.4, uncharacterized CH507-513H4.4; CH507-513H4.6, uncharacterized CH507-513H4.6; MT-ND3, mitochondrially encoded NADH dehydrogenase 3; FTL, ferritin light chain; PABPC1, poly(A) binding protein cytoplasmic 1; IGKC, immunoglobulin kappa constant; MALAT1, metastasis associated lung adenocarcinoma transcript 1

**Table S2 |** The clinicopathological characteristics of patients with esophageal squamous cell carcinoma (ESCC)

| Clinicopathologic characteristics | | Number of cases |
| --- | --- | --- |
| Age (years) | ≥ 60 | 51 |
|  | < 60 | 44 |
| Gender | Male | 54 |
|  | Female | 41 |
| Metastasis | Negative | 45 |
|  | Positive | 50 |
| Maxium diameter | ≤ 4.0 cm | 49 |
|  | > 4.0 cm | 46 |

**Table S3 |** qRT-PCR primer sequences

| Gene | Sequence |
| --- | --- |
| Human SOX17 | F: 5′-ACGCTTTCATGGTGTGGGCTAAG-3′ |
|  | R: 5′-GTCAGCGCCTTCCACGACTTG-3′ |
| Human MALAT1 | F: 5′-CAGCTCTGTGGTGTGGGATT-3′ |
|  | R: 5′-CTCACAAAACCCCCGGAAC-3′ |
| Human miR-199a | F: 5′-ACAGTAGTCTGCACATTGGTTA 3′ |
|  | Universal reverse primer |
| Human HIF1α | F: 5′-ACCTATGACCTGCTTGGTGC-3′ |
|  | R: 5′-GGCTGTGTCGACTGAGGAAA-3′ |
| Human U6 | F: 5′-UUGUGGAAAGGACGAAACACC-3′ |
|  | Universal reverse primer |
| Human GAPDH | F: 5′-ACAGTCAGCCGCATCTTCTT-3′ |
|  | R: 5′-GACAAGCTTCCCGTTCTCAG-3′ |
| Mouse SOX17 | F: 5′-GATGCGGGATACGCCAGTG-3′ |
|  | R: 5′-CCACCTCGCCTTTCACCTTTA-3′ |
| Mouse MALAT1 | F: 5′-GGGGGAATGGGGGCAAAATA- |
|  | R: 5′-AACTACCAGCAATTCCGCCA-3′ |
| Mouse miR-199a | F: 5′-CCCAGTGTTCAGACTACCTGTTC-3′ |
|  | Universal reverse primer |
| Mouse HIF1α | F: 5′-ACGACCACTGCTAAGGCATC-3′ |
|  | R: 5′-GTTTGTGCAGTATTGTAGCCAGG-3′ |
| Mouse U6 | F: 5′-CTCGCTTCACGAATTTGCGTGTCAT-3′ |
|  | Universal reverse primer |
| Mouse GAPDH | F: 5′-AGGTCGGTGTGAACGGATTTG-3′ |
|  | R: 5′-TGTAGACCATGTAGTTGAGGTCA-3′ |

Note: F, forward; R, reverse; SOX17, SRY-box transcription factor 17; MALAT1, metastasis associated lung adenocarcinoma transcript 1; HIF1α, hypoxia inducible factor 1 subunit alpha; GAPDH, glyceraldehyde-3-phosphate dehydrogenase.
