# Supplementary material for: SOX17-mediated MALAT1-miR-199a-HIF1α axis confers sensitivity in esophageal squamous cell carcinoma cells to radiotherapy
Source: Cell Death Discov. 2022 May 25;8:270. doi: 10.1038/s41420-022-01012-6 (PMC9132944; doi:10.1038/s41420-022-01012-6)
Supplement: Supplementary file 2 — Supplemental Materials - original western blots [file 41420_2022_1012_MOESM2_ESM.doc]

**Supplemental Materials - original western blots**

**Figure 2B**

**SOX17
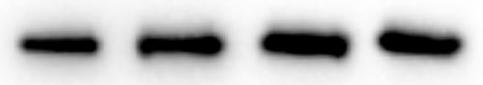
** **44 kDa**

**GAPDH
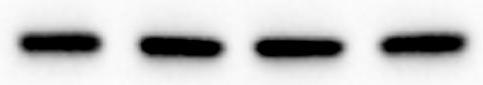
** **36 kDa**

**Figure 2D**

**0 Gy SOX17
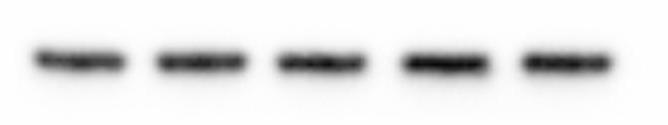
** **44 kDa**

**4 Gy SOX17
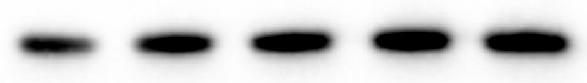
** **44 kDa**

**GAPDH
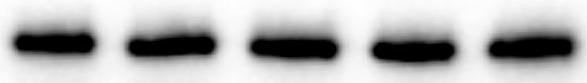
** **36 kDa**

**Figure 2F**

**SOX17
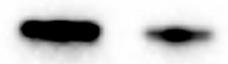
** **44 kDa**

**GAPDH
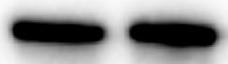
** **36 kDa**

**Figure 2H**

**SOX17
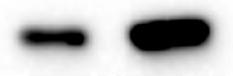
** **44 kDa**

**GAPDH
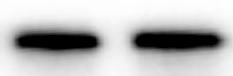
** **36 kDa**

**Figure 3J**

**SOX17
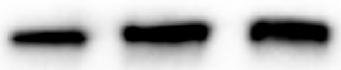
** **44 kDa**

**GAPDH
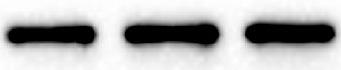
** **36 kDa**

**Figure 5F**

**HIF1α
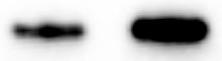
** **92 kDa**

**GAPDH
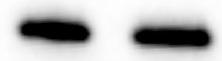
** **36 kDa**

**Figure 5K**

**HIF1α
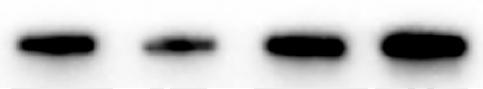
** **92 kDa**

**GAPDH
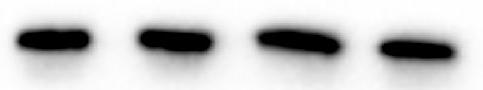
** **36 kDa**

**Figure 5O**

**HIF1α
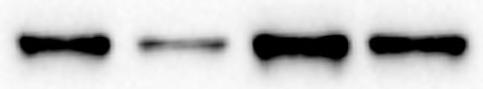
** **92 kDa**

**GAPDH
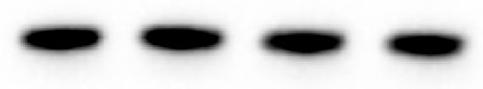
** **36 kDa**

**Figure 6B**

**SOX17
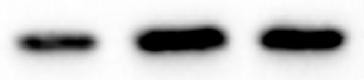
** **44 kDa**

**HIF1α
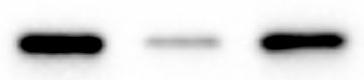
** **92 kDa**

**GAPDH
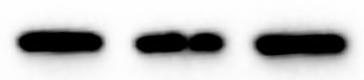
** **36 kDa**

**Figure 7E**

**SOX17
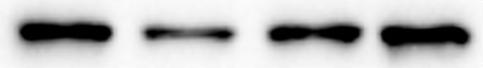
** **44 kDa**

**HIF1α
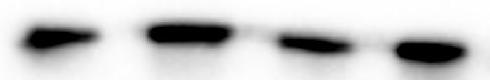
** **92 kDa**

**GAPDH
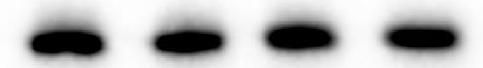
** **36 kDa**
